# Associating divergent lncRNAs with target genes by integrating genome sequence, gene expression and chromatin accessibility data

**DOI:** 10.1093/nargab/lqaa019

**Published:** 2020-03-20

**Authors:** Yongcui Wang, Shilong Chen, Wenran Li, Rui Jiang, Yong Wang

**Affiliations:** 1 Key Laboratory of Adaptation and Evolution of Plateau Biota, Northwest Institute of Plateau Biology, Chinese Academy of Sciences, Xining, Qinghai 810008, China; 2 Qinghai Provincial Key Laboratory of Crop Molecular Breeding, Northwest Institute of Plateau Biology, Chinese Academy of Sciences, Xining 810008, China; 3 MOE Key Laboratory of Bioinformatics, Bioinformatics Division, Beijing National Research Center for Information Science and Technology, Department of Automation, Tsinghua University, Beijing 100084, China; 4 CEMS, NCMIS, MDIS, Academy of Mathematics and Systems Science, Chinese Academy of Sciences, Beijing 100190, China; 5 Center for Excellence in Animal Evolution and Genetics, Chinese Academy of Sciences, Kunming 650223, China

## Abstract

Recent RNA knockdown experiments revealed that a dozen divergent long noncoding RNAs (lncRNAs) positively regulate the transcription of genes in cis. Here, to understand the regulatory mechanism of divergent lncRNAs, we proposed a computational model IRDL (**I**dentify the **R**egulatory **D**ivergent **L**ncRNAs) to associate divergent lncRNAs with target genes. IRDL took advantage of the cross-tissue paired expression and chromatin accessibility data in ENCODE and a dozen experimentally validated divergent lncRNA target genes. IRDL integrated sequence similarity, co-expression and co-accessibility features, battled the scarcity of gold standard datasets with an increasingly learning framework and identified 446 and 977 divergent lncRNA-gene regulatory associations for mouse and human, respectively. We found that the identified divergent lncRNAs and target genes correlated well in expression and chromatin accessibility. The functional and pathway enrichment analysis suggests that divergent lncRNAs are strongly associated with developmental regulatory transcription factors. The predicted loop structure validation and canonical database search indicate a scaffold regulatory model for divergent lncRNAs. Furthermore, we computationally revealed the tissue/cell-specific regulatory associations considering the specificity of lncRNA. In conclusion, IRDL provides a way to understand the regulatory mechanism of divergent lncRNAs and hints at hundreds of tissue/cell-specific regulatory associations worthy for further biological validation.

## INTRODUCTION

Most of the human genome is transcribed into RNAs but only a small fraction of them code for proteins, the rest are called noncoding RNAs (ncRNAs). Long noncoding RNAs (lncRNAs) are ncRNAs with lengths longer than 200 nt. Accumulating experimental studies have indicated that lncRNAs are versatile regulators of genes at the epigenetic, transcriptional and post-transcriptional levels (1,2), and their alterations and dysfunctions have been associated with some complex diseases, including cancer (3–6). However, the vast majority of lncRNAs are functionally uncharacterized, and the molecular mechanism through which they act remains unclear. Thus, identifying the functional lncRNAs and then inferring the biological process in which they participate represent the major challenges in understanding genome complexity and RNA-mediated gene regulation (7).

Many methods have been proposed to categorize lncRNAs and characterize their function based on genome resources, including genome distribution, expression pattern, chromatin features, and subcellular localization (8–11), and initial evidence supports the coexpression of tissue-specific lncRNAs and protein-coding genes. However, there has been debate for years as to whether the expression of lncRNAs correlates with nearby (*cis*) or distal (*trans*) protein-coding genes, and whether lncRNAs regulate nearby genes (8,9,12–14). In addition, Yin *et al.*, (15) revealed the opposing role of the lncRNA Haunt in regulating HOXA gene expression during embryonic stem cell (ESC) differentiation: the Haunt DNA locus contains potential HOXA enhancers. The Haunt transcript binds to chromatin and acts to prevent the expression of HOXA (15). These findings suggest a complicated mechanism of lncRNA regulation.

Recently, Luo *et al.*, (7) has provided a novel way to study the function of lncRNAs and their regulatory mechanism. They categorized lncRNAs into different biotypes via a comprehensive genome locus, and revealed that divergent lncRNAs (transcribed in the opposite direction to nearby protein-coding genes) had a non-random distribution in genome. Among 24 randomly selected divergent lncRNAs that were successfully knocked down by RNAi (RNA interference) experiments, depletion of 75% (12 of them are transcription factors) led to downregulation of nearby protein-coding genes, which indicates that at least a subset of lncRNAs positively regulate the transcription of genes in *cis*, and that their function could be inferred through the role of neighboring genes (7). However, only a dozen regulations were confirmed in (7), and the authors only discussed the regulatory mechanism for one mouse gene: Evx1(7). Thus, the fundamental role of divergent lncRNAs in gene regulation is urgently needed to assess the effects of lncRNAs on gene expression and increase the number of functionally characterized lncRNAs.

Here, we designed a computational model, called IRDL (Identify the Regulatory Divergent LncRNAs), to understand the fundamental molecular mechanism in divergent lncRNA regulation and construct a comprehensive divergent lncRNAs regulatory network. Specifically, the data sources that were previously used to characterize lncRNA function (8–10), including genome sequence, expression and chromatin accessibility, were incorporated to learn the genomic properties for divergent lncRNA-gene regulatory relationships. Using the knowledge of experimentally validated regulations and an increasingly learning framework, IRDL identified 446 and 977 regulatory divergent lncRNA and gene associations for mouse and human, respectively. The functional analysis indicates that the divergent lncRNA strongly correlates with essential developmental regulatory genes. Those genes usually have transcription factor activity, and are involved in immune- and cancer-related pathway. Importantly, the predicted loop structure validation and canonical database search offer a possible regulatory mechanism of divergent lncRNA: the divergent lncRNA DNA locus contains potential gene enhancers, and the RNA transcript is crucial to recruit the mediated complex to help gene transcript. Furthermore, the specificity of lncRNA leads to the tissue/cell-specific regulatory association, which offers a great opportunity to validate the novel predictions in certain circumstances. IRDL is freely available at https://github.com/wangyc82/IRDLv1.

## MATERIALS AND METHODS

### IRDL framework

**Figure 1.**
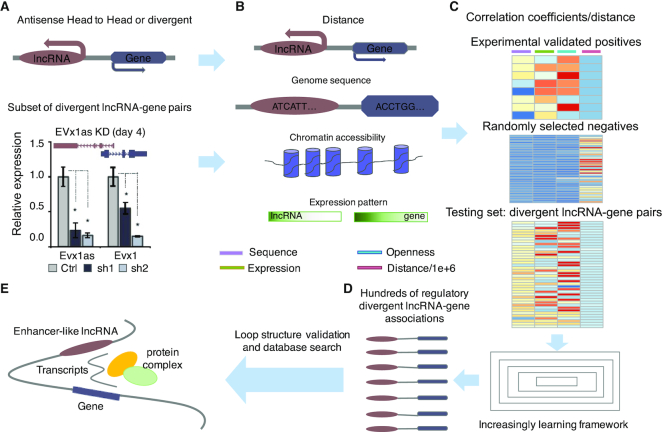
The framework of IRDL. (**A**) The experimentally validated divergent lncRNA-gene regulations. (**B**) The genome resources that were introduced to model divergent lncRNA regulation. (**C**) The genome sequence, expression and chromatin accessibility correlation coefficients and genome distance were calculated on experimentally validated lncRNA-gene pairs, randomly selected nearby lncRNA-gene pairs and divergent lncRNA-gene pairs for training positives, negatives and testing set, respectively. (**D**) An increasing learning framework was proposed to unveil the regulatory divergent lncRNA-gene pairs from the testing set. (**E**) The potential molecular mechanism was elucidated from the predicted loop structure validation and database search.

Previous work has validated a dozen regulatory relationships between divergent lncRNAs and genes through RNA knockdown experiments (Figure 1A), which encourage us to collect several genome resources, including genome distance, genome sequence, expression and chromatin accessibility, for detecting the molecular mechanism behind that regulatory phenomenon (Figure 1B). Through introducing genome sequence, expression and chromatin accessibility correlation coefficients and genome distance on experimental validated regulatory lncRNA-gene pairs (positives), randomly selected nearby lncRNA-gene pairs (negatives) and divergent lncRNA-gene pairs (testing set) into an increasing learning framework (Figure 1C), hundreds of regulatory divergent lncRNA-gene associations were uncovered from the testing set (Figure 1D). The predicted loop structure validation and canonical database search indicate a possible regulatory mechanism of divergent lncRNA: the divergent lncRNA DNA locus contains potential gene enhancers, and the RNA transcript is crucial to recruit the mediated complex to help gene transcript (Figure 1E). We explain the above framework in detail in the following subsections.

### Increasingly learning framework

**Figure 2.**
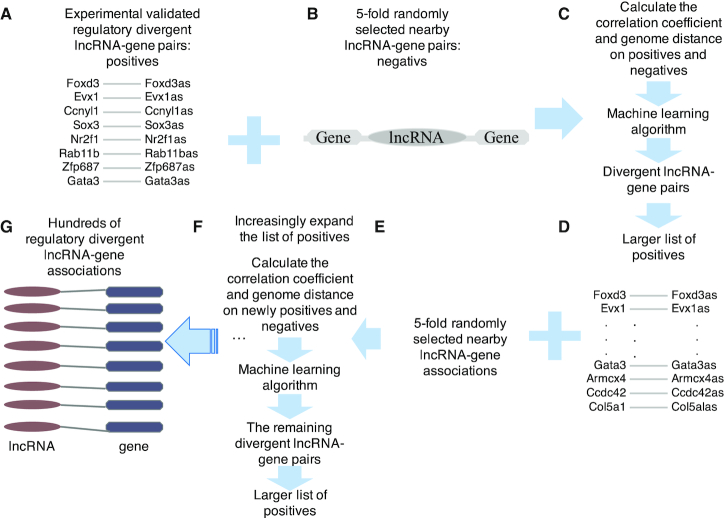
The increasingly learning framework. (**A**) The training positives: experimentally validated divergent lncRNA and gene regulations. (**B**) The training negatives: 5-fold randomly selected nearby lncRNA-gene pairs. (**C**) The machine learning algorithm that is taken validated regulatory lncRNA-gene pairs as training positives, 5-fold randomly selected nearby lncRNA-gene pairs as the training negatives and divergent lncRNA-gene pairs as the testing set, was introduced to generate the larger list of positives. The genome sequence, expression and chromatin accessibility correlation coefficients and genome distance were used as input features for that machine learning algorithm. (**D**) The increased regulatory divergent lncRNA-gene pairs generated from the machine learning algorithm. (**E**) Another 5-fold randomly selected nearby lncRNA-gene pairs. (**F**) The iterative process to generate the larger list of positives with genome sequence, expression and chromatin accessibility correlation coefficients and genome distance as the input feature for newly positives and negatives. (**G**) Hundreds of regulatory divergent lncRNA-gene associations generated via increasingly expanding the list of positives.

To learn the molecular mechanism in divergent lncRNA regulation from experimentally validated regulatory associations and generate more regulatory associations, an increasingly learning framework was proposed. Specifically, eight experimentally validated regulatory pairs of divergent lncRNAs and genes were introduced as the training positives (Figure 2A), and the randomly selected 5-fold nearby lncRNA-gene pairs were introduced as the training negatives (Figure 2B). The genome distance, and sequence, expression and openness (chromatin accessibility) correlation coefficients were calculated for training positives and negatives, which were utilized as the input feature for training a support vector machine (SVM) classification model (Figure 2C). Then, the divergent pairs of lncRNAs and genes with genome distance, and sequence, expression and openness correlation coefficients as their representative feature were predicted by that SVM classification model. Those divergent lncRNA-gene pairs with SVM predicted score larger than 0.9 were added into the list of training positives (Figure 2D), and another randomly selected nearby lncRNA-gene pairs were collected as the training negatives (Figure 2E). The genome distance, sequence, expression and openness correlation coefficients were calculated as the representative feature for newly training positives and negatives, which were utilized to train a new SVM classification model (Figure 2F). The remaining divergent lncRNA-gene pairs with genome distance, and sequence, expression and openness correlation coefficients as the representative feature were predicted by this newly trained SVM classification model. Then, the divergent lncRNA-gene pairs with SVM predicted score larger than 0.9 were added into the list of training positives, and another randomly selected 5-fold nearby lncRNA-gene pairs were collected as the training negatives (Figure 2F). The remaining divergent lncRNA-gene pairs were predicted, and the pairs with predicted score larger than 0.9 were added into the list of training positives (Figure 2F). The procedure was executed until only one new positive was added in two continuous steps. At last, the regulatory divergent lncRNA and gene associations were unveiled for further analysis (Figure 2G).

The SVM classification model (motivated by statistical learning theory (16,17)) is the key for implementation of the IRDL model. It was run via the R ‘e1071’ package. The model parameters, including the penalty parameter and the RBF kernel parameter, were determined by 3-fold cross-validation. To avoid an extreme imbalance of positives and negatives, the ratio of training positives and negatives was set to 1:5. Thus, the classification problem in each step of increasing learning framework was imbalanced. To evaluate the classification model on imbalance problem, the area under the precision-recall curves (AUPR), a better index for evaluatiing the imbalance problem (18), was introduced. The AUPR was calculated via the R ‘PRROC’ package.

### Definition of the chromatin accessibility value: openness, and the genome distance

The score to quantify the chromatin accessibility (i.e. openness) for the lncRNAs and genes was introduced by (19). To ensure that the score could be comparable across different conditions and remove the sequencing depth effect, for a certain region of length, the openness score was defined as the fold change of the number of reads per base pair and was calculated as follows: $\mathrm{O} = \frac{(X+\delta )/L}{(Y+\delta )/L_{0}}$, where *X* is the count of reads in the region with length *L*, *Y* is the count of reads in a background region with length *L*_0_ and δ is a pseudocount (the default value of is 5 in our setting). The count reads were obtained from the DNase-seq deposited in ENCODE (20).

The genome distances between lncRNAs and genes were calculated to display the co-localization: if they were located in the same chromatin, the distance defined by the start site of gene minus the start site of lncRNA, and if they were located in different chromatin, the distance defined as the infinity.

### Prediction of loop structure for regulatory associations based on DeepTACT

To detect whether there are physical interactions among IRDL-identified regulatory lncRNA-gene pairs, the DeepTACT method that was developed to predict high-resolution chromatin contacts based on genome sequence and bootstrapping deep learning (21), was introduced. It was applied on the genome sequences for the ‘promoter’ region of lncRNA/gene (2000 bp before lncRNA/gene start locus). Overall, there are over 10% mouse or human regulatory associations with the loop structure. To show the significance of above results, a significance test was performed. That is, 20-sampled groups, each containing 977 random human promoter–promoter pairs (same number of regulatory human lncRNA-gene pairs) were scored with the DeepTACT model trained in tB (total B cells) and the number of pairs that were predicted as positive (loop structure) were counted. The number of pairs that were predicted to be positives is ∼36–58, which is significantly smaller than divergent cases (104).

### Correlation analysis, statistical test and regression model

The correlation analysis on divergent lncRNA-gene pairs and randomly selected neighboring lncRNA-gene pairs was performed through calculating Pearson correlation coefficients (PCCs) based on genome data sources. Specifically, when lncRNA and gene were represented either by expression or chromatin accessibility, the PCCs were calculated based on the expression or chromatin accessibility profile. When lncRNA and gene were represented by genome sequence, the 3-mer sequence feature (an 84-dimensional vector with the first four elements as the frequency of each type of nucleic acid, the following 16 elements as the frequency of all possible combinations of two types of nucleic acid and the last 64 elements as the frequency of all possible combinations of three types of nucleic acid) was first applied to represent lncRNA/gene, and the PCCs were calculated based on their 3-mer sequence features. The statistical test for the significance of differences among different types of correlations was based on the KS-test, which was performed using R.

The Random Forest (RF) regression model was introduced here for comparison. Specifically, we run RF regression model with gene as the response, lncRNAs as the regulators and the expression level as the regression feature. The regulatory lncRNAs were determined from the feature importance that was generated from the RF model. The RF regression model was performed via the R ‘randomForest’ package with default parameters.

### Identification of tissue/cell-specific regulations

To identify the tissue/cell-specific regulations, we checked the expression of given regulatory lncRNA-gene associations, and determined whether both lncRNA and gene involved in that regulatory association are specifically expressed in a certain type of tissue/cell. That is, for each regulatory lncRNA-gene pair, reported tissues/cells that had samples with high-gene expression levels (larger than six), and tissues/cells that had samples with high-lncRNA expression levels (larger than four), respectively. Then, an enrichment score for each overlap tissue/cell was defined as follows: $\mathrm{ES} = \frac{m \times n}{p \times q}$, where *m* and *n* are the number of samples with high-expression levels belonging to a certain type of tissue/cell for gene and lncRNA, respectively, and *p* and *q* are the number of all samples with high-expression levels for gene and lncRNA, respectively, and the final specific tissue/cell for each lncRNA-gene regulation is assigned by the tissue/cell with largest score.

### Material

The gold standard positives for the IRDL model were collected from (7): the eight regulatory divergent lncRNA-gene associations that were validated by RNA knockdown experiments (Figure 2A). The candidates for regulatory associations were those mouse and human divergent pairs of lncRNAs and genes, which were also generated from (7). In (7), the authors generated 1665 and 2847 pairs of divergent lncRNAs and genes for mouse and human, respectively, and there are 650 mouse and 1369 human divergent pairs with all genome data sources available, including the genome sequence, expression and chromatin accessibility. The randomly selected nearby pairs of lncRNAs and genes (exclusive from the candidates) were used as the training negatives for IRDL. The genome sequence for lncRNA and gene and their promoter region came from the mouse mm9 and human hg19 genome sequence, respectively, which were downloaded from UCSC Genome Browser. The paired gene expression and chromatin accessibility data were from ENCODE and we used a diverse panel of cell lines with both expression and accessibility data. This includes 56 cell lines in the case of mouse and 148 cell lines in the case of human (https://www.pnas.org/content/suppl/2018/07/07/1805681115.DCSupplemental). The details of the dataset were from our previous publication in (19). The divergent lncRNAs proposed in (7) were presented by their official gene symbols. The NONCODE ID was used to represent the lncRNAs in ENCODE RNA-seq and DNase-seq project. Thus, the NONCODE ID conversion (22) was applied to convert the gene symbol into NONCODE ID. The information for biosamples was also collected from ENCODE. The log2 transformation was applied to the expressions for both lncRNAs and genes before calculating correlation coefficients.

## RESULTS

### The correlation analysis on experimentally validated regulations hints at the clear genomic properties in regulatory lncRNA-gene associations

**Figure 3.**
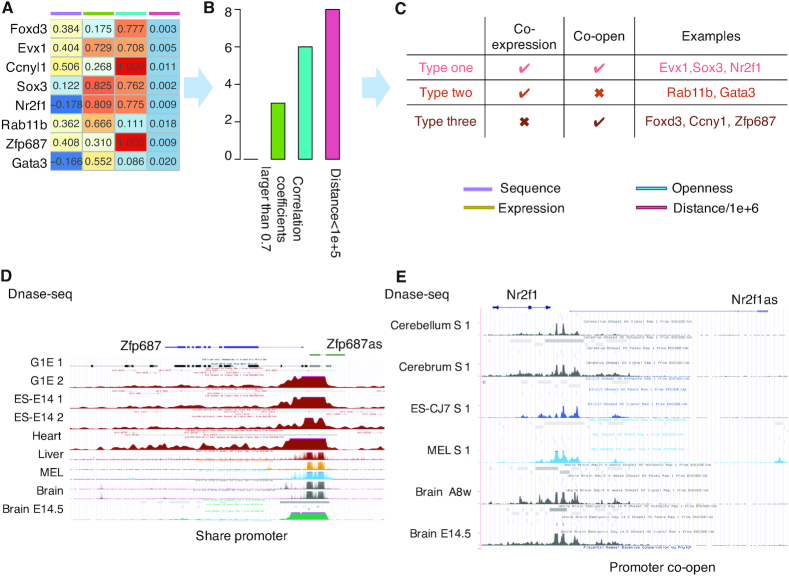
The differences in genome profiles of experimentally validated regulatory lncRNA-gene pairs. (**A**) The genome sequence, expression, and chromatin accessibility correlation coefficients and genome distance among validated regulatory lncRNA-gene pairs. (**B**) The number of validated regulatory lncRNA-gene pairs with clear genomic properties, including high-correlation coefficients (>0.7) and closely located (<100 kb). (**C**) The summary of genomic properties among those validated regulatory lncRNA-gene pairs in terms of correlation and genome distance. (**D** and **E**) Examples showing how regulatory lncRNA-gene pairs are correlated in terms of chromatin accessibility: the mouse gene Zfp687 shares the common promoter locus with its nearby divergent lncRNA (D); the promoter region of the mouse gene N2f1 and its nearby divergent lncRNA are co-open (E).

Luo *et al.*, (7) constructed 1665 and 2846 pairs of divergent lncRNAs and nearby protein-coding genes for mouse and human, respectively, and validated 18 positively regulatory relationships among 24 randomly selected mouse pairs (7). We took a close look at those validated regulations in terms of genomic properties (including genome sequence, expression and chromatin accessibility), especially for those lncRNAs neighboring a transcription factor (TF) gene (Figure 3A). The correlation analysis indicates the obvious genomic properties among those regulatory lncRNA-gene pairs. There are eight lncRNA-gene pairs with all three genome data sources available, and none of them show a close correlation relationship in terms of genome sequence. Five of them strongly correlated in expression, and six of them closely correlated in chromatin accessibility (Figure 3B). Eight lncRNA-gene pairs correlated either in terms of expression or chromatin accessibility (Figure 3C), and the highest chromatin accessibility correlation coefficients hint at the important role of chromatin accessibility in divergent lncRNA regulation. DNase-seq profile for mouse genes Zfp687 and N2f1 display two ways in which the nearby lncRNA-gene pairs share the same chromatin accessibility profiles: they either share the common promoter locus or their promoter regions are co-open. Together, these results suggest that, the regulatory lncRNA-gene pairs displayed several clear genomic properties (such as high correlation in terms of expression and chromatin accessibility, and close positioning). Based on these properties, the searching space of functional lncRNAs with known target genes could be expanded.

Inspired by that, we proposed an increasingly learning framework to identify the regulatory lncRNA-gene pairs from those divergent lncRNA-gene pairs proposed in (7). Learning from the knowledge of experimentally validated regulatory lncRNA-gene pairs, the increasingly learning framework identified 446 and 977 lncRNA-gene pairs as regulatory associations for mouse and human, respectively (Supplementary Figure S1A). To validate these identified regulatory associations, three evaluations were implemented, including comparison of genomic properties with experimentally validated regulations, predicted loop structure validation, and canonical database checking. Moreover, to suggest the possible function of those regulatory divergent lncRNAs, functional and pathway enrichment analyses were performed on their target genes.

### IRDL generated regulatory divergent lncRNA-gene associations that exhibit similar genomic properties to experimentally validated ones

**Figure 4.**
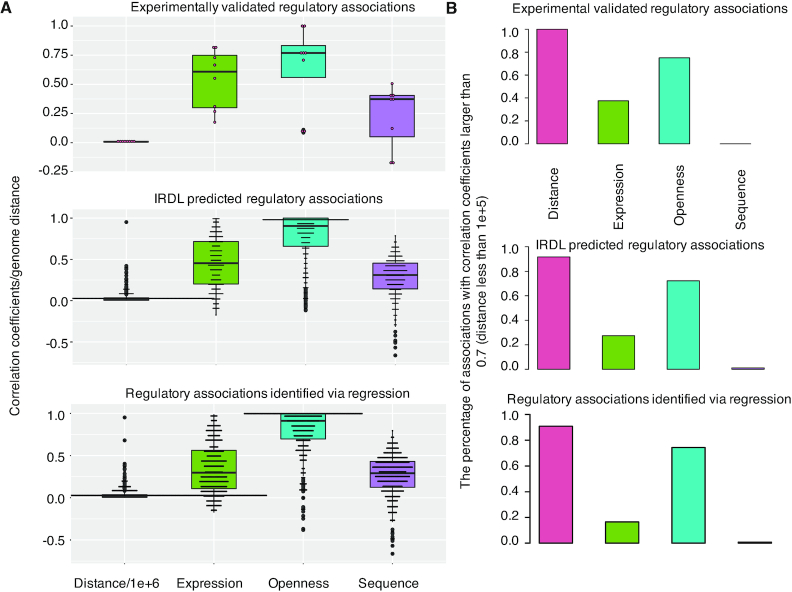
The comparison of genomic properties on IRDL-identified mouse regulations with the experimentally validated ones and the ones identified from the regression model. (**A**) The genome sequence, expression and chromatin accessibility correlation coefficients and genome distance on experimentally validated regulations, IRDL-identified ones and the ones revealed from the RF regression model. (**B**) The number of lncRNA-gene pairs with high-correlation coefficients (>0.7) and close genome distance (<100kb).

We first tested whether the IRDL could generate regulatory associations that have similar genomic properties to experimentally validated ones. The comparison of correlation coefficients and genome distance on mouse 446 regulatory associations indicate that IRDL generates regulations sharing similar genomic properties to experimentally validated ones, including close positioning, poor correlation in genome sequence, high correlation in expression and chromatin accessibility and is more closely related in terms of chromatin accessibility (Figure 4A). Furthermore, when compared with the regression model based on expression, the regulations identified through IRDL obtained much similar properties to experimentally validated ones. That is, they displayed higher expression and openness correlation coefficients (Figure 4A), and there were many more identified regulatory associations with expression and openness correlation coefficients >0.7 (Figure 4B). The comparison of genomic properties on human 977 regulatory associations tells us a similar story. That is, compared with the regression model, IRDL identified divergent lncRNAs that were more correlated with their regulated genes in terms of expression and chromatin accessibility (Supplementary Figure S2A). Furthermore, they were more strongly related in chromatin accessibility than in expression pattern (Supplementary Figure S2B). These results together indicate that IRDL did identify the regulatory associations exhibiting similar genomic properties to experimentally validated ones, and the regulatory associations are worthy for further discussion. Moreover, the function of those divergent lncRNAs could be inferred from their regulated genes.

### Divergent lncRNAs are prone to regulate genes linking with developmental regulatory processes and transcription factor activity

**Figure 5.**
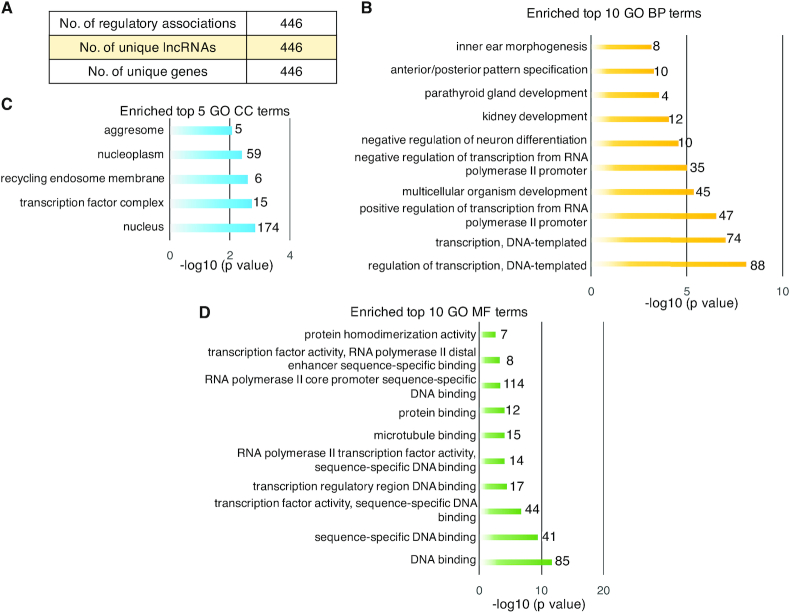
The enriched functions on mouse genes that were identified by IRDL to be the regulatory target of divergent lncRNAs. (**A**) The summary of IRDL-identified regulatory relationships. (**B**) The enriched top 10 GO biological process (BP) terms. (**C**) The enriched top 5 GO cellular component (CC) terms. (**D**) The enriched top 10 GO molecular function (MF) terms. The number shows the number of genes.

The IRDL model identified 446 and 977 regulatory divergent lncRNA and gene associations for mouse and human, respectively, which contain the 446 and 977 unique mouse and human genes and lncRNAs, respectively (Figure 5A and Supplementary Figure S3A). The genes regulated by lncRNA offer a way to understand the function of lncRNA. Thus, to decipher the lncRNA function, the function and KEGG pathway enrichment analysis on genes that were predicted to be the regulatory target of divergent lncRNA were introduced here. DAVID bioinformatics resources reports that these mouse genes are closely related to organ developmental regulatory processes (such as kidney, parathyroid gland and multicellular organism development, Figure 5B) and transcription factor activity (Figure 5D), and most of them are located in the nucleus (Figure 5C). The KEGG pathway analysis indicate that those genes prefer to participate in immune-related KEGG pathways (MAPK signaling, Notch signaling and HTLV-I infection) and the cardiovascular disease pathway. For human genes that are predicted to be regulatory targets of divergent lncRNAs, the developmental regulatory processes (such as lung, palate development, Supplementary Figure S3B) and transcription factor activity (Supplementary Figure S3D) are also connected to them. Most of them are located in the nucleus (Supplementary Figure S3C). The stem cell regulation and cancer-related KEGG pathways are related with them. These results together indicate the important regulatory function of divergent lncRNA target genes, which suggests the crucial regulatory role of those divergent lncRNAs in organ development, the immune system and cancer.

### The predicted loop structure validation and canonical database search indicate a possible regulatory mechanism of divergent lncRNA

The previous correlation analysis indicates the similar genomic properties of IRDL-identified regulatory associations to experimentally validated ones. To further validate these regulatory associations and explore the regulatory mechanism of divergent lncRNAs, the predicted loop structure validation and canonical database search were implemented.

**Figure 6.**
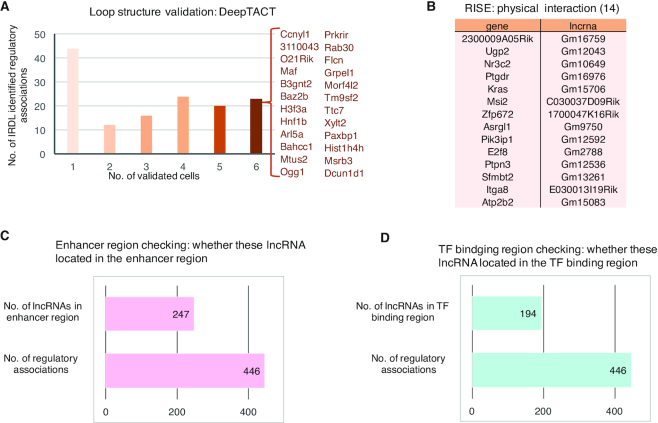
The validations on IRDL-identified mouse divergent lncRNA-gene regulations. (**A**) The predicted loop structure validation results on IRDL-identified mouse regulations. The associations validated by all six cell conditions are highlighted. (**B**) The physical interactions validated from RISE for IRDL-identified mouse regulations. (**C**) The number of mouse regulatory divergent lncRNAs identified by IRDL, which is located in the mouse enhancer region. (**D**) The number of mouse regulatory divergent lncRNAs identified by IRDL, which is located in the mouse TF binding region.

The high-throughput chromosome conformation capture (Hi-C) technique is a powerful tool for studying the spatial organization of chromatin in a cell, as it quantifies physical interactions between all possible pairs of fragments simultaneously (23). However, due to sequencing cost, most available Hi-C datasets have relatively low resolution, such as 25 or 40 kb (24). Researchers have developed computational approaches to infer high-resolution Hi-C interaction matrices from low-resolution Hi-C data based on deep convolutional neural network (21,25). Here, the divergent lncRNA and gene pairs proposed in Luo *et al.*, (7) , are close positioning in genome (usually located within 10 kb). Thus, to detect the possible loop structure among IRDL-identified regulatory associations, the computational model (DeepTACT) was introduced (21). Over 10% of mouse or human regulatory interactions were predicted to have a loop structure under at least one cell condition, and over 5% received six cell supports (Figure 6A and Supplementary Figure S4A). To further check the physical interaction of IRDL-identified regulatory associations, RISE, a comprehensive repository of RNA–RNA interactions in three species (mouse, human and yeast) that came from transcriptome-wide sequencing-based experiments (such as PARIS, SPLASH, LIGRseq and MARIO), targeted studies (such as RIAseq, RAP-RNA and CLASH), and primary databases and publications (26), was introduced. As a result, 14 mouse and 6 human regulatory associations were confirmed in RISE (Figure 6B and Supplementary Figure S4B). All these results support that physical interaction might exist among those regulatory associations.

Previous studies have suggested a possible mechanism of lncRNA in the regulation of neighboring genes: activating RNAs are transcribed from enhancer-like lncRNAs and are required for recruitment of the protein complex to bridge enhancer-like lncRNAs and the promoter of a coding gene (27). The predicted loop structure validation and RISE database search seem to indicate the physical interaction of IRDL-identified regulatory associations. To validate whether those divergent lncRNAs would act as enhancer-like lncRNAs, the enhancer and TF binding region were checked. As a result, over 50% (247/446) of mouse regulatory divergent lncRNAs share genome loci with mouse enhancers (Figure 6C), and approximarely 25% (236/977) of human regulatory divergent lncRNAs share genome loci with human enhancers (Supplementary Figure S4D). Furthermore, over 40% (194/446) of mouse regulatory divergent lncRNAs share genome loci with the mouse TF binding region (Supplementary Figure 6D), and approximately all human regulatory divergent lncRNAs share genome loci with the human TF binding region (Supplementary Figure S4D). These results suggest that the regulatory divergent lncRNAs identified by IRDL play similar roles as eRNA and regulate transcription from bridging scaffold model.

Then, we attempted to find evidence for our regulatory associations from canonical databases specifically designed for the current experimentally validated lncRNA regulatory interactions. Because most of these databases only deposit human regulatory interactions, such as EVLncRNAs and LongHorn, only human regulatory associations were analyzed here. EVLncRNAs collects all published experimentally validated lncRNAs of different species, including animals, plants and microbes (28), and eight IRDL human regulatory associations were found in EVLncRNAs (Supplementary Figure S4C). LongHorn predicts modulation of canonical regulators (or effectors) in human, including miRNA, RBP and TF, by lncRNAs (29), and more than 25% (273/977) of IRDL human regulatory associations were found in LongHorn (Supplementary Figure S4E). In addition to EVLncRNAs and LongHorn, the Genotype-Tissue Expression (GTEx) project, which is a comprehensive public resource to study the relationship between genetic variation and gene expression in multiple human tissues, was also introduced for validation. As a result, 33 genetic variations in the divergent lncRNA locus linked with the expression of IRDL-identified regulatory target genes. That is, the research on canonical databases provides support for a subset of IRDL-identified regulatory associations, which offers confidential candidates for further experimental validation.

### A tissue/cell specific regulatory network offers a great opportunity to understand the function of divergent lncRNAs in a certain circumstance

In comparison with protein-coding genes, the major difference in lncRNAs lies in the fact that lncRNAs have much more tissue/cell-specific patterns (30–32). Thus, we attempted to specify our regulatory associations as tissue/cell-specific ones. The number of IRDL-identified regulatory associations in the same mouse and human tissue/cell is shown in Supplementary Figure S5. The brain is the tissue/cell type that most mouse regulatory associations are specific to, and blood is the majority tissue/cell type that most human regulatory associations are specific to.

Because we obtained tissue/cell-specific regulations, we first sought to examine experimentally validated regulations in Luo *et al.*, (7). Except for Evx1 and Gata3as with low expression levels in all bio-samples, the remaining six regulations were specific to a tissue/cell type (Supplementary Table S1). Five regulations were found in brain tissue. For instance, Sox3 (SRY-Box 3) was reported to be related to neural stem cell maintenance (33), and Nr2f1 (The nuclear orphan receptor COUP-TFI) was important for the differentiation of oligodendrocytes (34), suggesting that the regulator Sox3as and Nr2f1as have a great chance of being involved in brain development. In addition, Ccny1as, Zfp687as and Gata3as share genome loci with the mouse enhancer region, and Zfp687as and Gata3as share loci with the mouse TF binding region. These results suggest that the bridging scaffold model may be involved in regulatory divergent lncRNAs.

Then, we listed the details for the top ten IRDL predictions (the predictions were arranged according to the IRDL prediction score) in Supplementary Tables S2 and 3 for mouse and human, respectively. Except for one human prediction (JAZF1-JAZF1AS1), the remaining mouse and human predictions have physical interactions supported either by loop structure validation or by RISE. Eight of the top ten human predictions obtained database support evidence, suggesting that our predictions are worthy for further experimentally validated. Importantly, four mouse predictions and two human predictions obtained both enhancer and TF binding region evidence, confirming the assumption of a bridging scaffold model for divergent lncRNAs. All these results indicated that, IRDL provides tissue/cell-specific regulatory associations between divergent lncRNAs and neighboring genes. The function and pathway analysis on genes that were predicted to be the target of divergent lncRNAs provide an excellent opportunity to understand the function of those lncRNAs in a certain circumstance. Importantly, the predicted loop structure validation and canonical database search hint at the bridging scaffold model of divergent lncRNAs.

## DISCUSSION

The previous study suggested that divergent lncRNAs, or at least a subset of them, positively regulated the transcription of genes in *cis* and participated in biological processes in which nearby genes were involved (7). To identify more divergent lncRNAs with known regulatory targets and expand the subset of functionally characterized lncRNAs, a novel computational model, called IRDL, was proposed. It first learned the genomic properties of regulatory associations validated by RNA knockdown experiments, and then expanded the regulatory associations through an increasingly learning framework based on those identified genomic properties. IRDL was applied to the mouse and human divergent pairs of lncRNAs and genes that were generated in (7). As a result, IRDL identified hundreds of regulatory lncRNA-gene associations, which share similar properties to experimentally validated ones, including close positioning (over 90% <100 kb), poor correlation in genome sequence, high-correlation in expression and chromatin accessibility, and more closely related chromatin accessibility. The correlation analysis on experimentally validated and IRDL-identified regulatory associations hint at the crucial role of expression pattern and chromatin accessibility in modeling divergent lncRNA regulation. In addition, ∼95% (431/446) of mouse and 97% (951/977) of human regulatory associations revealed from IRDL were positively correlated with openness, and 93.4.9% (417/446) of mouse and 83.1% (811/977) of human regulations were positively correlated with expression (Supplementary Figure S1B). This finding suggests that positive relationships might be involved in established regulatory associations. The function and pathway analysis on genes regulated by lncRNAs indicate the developmental regulation and transcription factor activity of regulatory divergent lncRNAs. Nearly all top ten mouse and human predictions were obtained support evidence from physical interaction, and some of them received both enhancer and TF binding region evidence. This indicates the bridging scaffold regulatory model of divergent lncRNA. Importantly, most of the top predictions obtained database evidence, indicating that the predictions are worthy for further validation. In conclusion, IRDL battles the scarcity of gold standard dataset with an increasingly learning framework, and generates hundreds of regulatory lncRNA-gene associations; reveals that the expression and chromatin accessibility are linked to divergent lncRNA regulations; suggests a possible way to understand the regulatory mechanism of lncRNA; provides tissue/cell-specific confidential candidates for further experimental validation.

The SVM classification model was introduced to train the classification model in the IRDL increasingly learning framework. In addition to SVM, there are many machine learning classification models, such as RF, neural network and deep learning. RF was proven to have the state-of-the-art performance as did as SVM among all machine learning algorithms, except for deep learning (35). Deep learning outperforms the state-of-the-art machine learning models but requires a considerable number of samples to learn hundreds of thousands of model parameters (36). Because we only had thousands of lncRNA-gene pairs, only RF was chosen for comparison. Specifically, the genome data correlation coefficients among mouse and human divergent lncRNA-gene pairs and 5-fold randomly selected nearby lncRNA-gene pairs were used to train the RF classification model, and the 5-fold cross-validation was applied to assess the performance of the models. The higher AUPR (a better index for evaluation of the imbalance problem (18)) shows that SVM performs better than RF (Supplementary Figure S6). Thus, the SVM classification model was introduced here as the classification model.

Here, the genome features, including genome sequence, expression and chromatin accessibility, which had been used to characterize lncRNAs’ function, were introduced to associate divergent lncRNAs with protein-coding genes. In addition, other features have also been previously used to link lncRNAs with their function, such as subcellular localization (11). The co-localization was already introduced here by defining the genome distance between lncRNA and gene. The analysis indicates that both experimentally validated and IRDL-identified regulatory lncRNA-gene associations are close positioning, and most of them (90%) were locate within 100 kb in genome. Thus, the co-localization is an important property of divergent lncRNA regulation, and with the complementation of expression and chromatin accessibility, more confidential candidates that are worthy for further experimental validation could be generated.

## Supplementary Material

lqaa019_Supplemental_File
