# Supplementary material for: Associating divergent lncRNAs with target genes by integrating genome sequence, gene expression and chromatin accessibility data
Source: NAR Genom Bioinform. 2020 Mar 20;2(2):lqaa019. doi: 10.1093/nargab/lqaa019 (PMC7671357; doi:10.1093/nargab/lqaa019)
Supplement: lqaa019_Supplemental_File [file lqaa019_supplemental_file.docx]

Supplementary Materials

**Associating divergent lncRNAs with target genes by integrating genome sequence, gene expression, and chromatin accessibility data**

Yongcui Wang^1,2*^, Shilong Chen^1^, Wenran Li^3^, Rui Jiang^3^, Yong Wang^4,5*^

^1^ Key Laboratory of Adaptation and Evolution of Plateau Biota, Northwest Institute of Plateau Biology, Chinese Academy of Sciences, Xining, Qinghai, 810008, China

^2^ Qinghai Provincial Key Laboratory of Crop Molecular Breeding, Northwest Institute of Plateau Biology, Chinese Academy of Sciences, Xining, 810008, China

^3^ Ministry of Education Key Laboratory of Bioinformatics, Bioinformatics Division, Department of Automation and TNLIST, Tsinghua University, Beijing 100084, China

^4^ CEMS, NCMIS, MDIS, Academy of Mathematics and Systems Science, Chinese Academy of Sciences, Beijing, 100190, China

^5^ Center for Excellence in Animal Evolution and Genetics, Chinese Academy of Sciences, Kunming, 650223, China

* To whom correspondence should be addressed. Email: ycwang@nwipb.cas.cn; ywang@amss.ac.cn.

**Content:**

**Table S1**

The details for experimentally validated regulations, including the specific tissue, enhancer region and TF binding region checking.

**Table S2**

The details for top ten mouse predictions, including the specific tissue, enhancer region and TF binding region checking, and curated database evidences.

**Table S3**

The details for top ten mouse predictions, including the specific tissue, enhancer region and TF binding region checking, and curated database evidences.

**Figure S1**

The data summary for mouse and human data, and the genome sequence, expression, and chromatin accessibility correlation coefficients on IRDL identified mouse and human regulations.

**Figure S2**

The comparison of genomic properties on IRDL identified human regulations with experimentally validated ones and RF model revealed ones. A, the genome sequence, expression, and chromatin accessibility correlation coefficients and genome distance on experimentally validated regulatory associations, IRDL identified regulations, and the regulations revealed from RF regression model. B, the number of lncRNA-gene pairs with high correlation coefficients (larger than 0.7) and close genome distance (less than 10kb.

**Figure S3**

The enriched functions and pathways on human genes that were identified by IRDL to be regulatory target of the divergent lncRNAs. A, the summary of IRDL identified regulatory relationships. B, The enriched top 10 BP terms. C, The enriched top 5 GO CC terms. D, The enriched top 10 GO MF terms. E, The enriched top 5 KEGG pathways. The numbers on the bar highlight the number of genes with that enriched terms.

**Figure S4**

The validations on IRDL identified human divergent lncRNA regulations. A, the loop structure validation results on IRDL identified human regulations. B, the physical interactions validated from RISE for IRDL identified human regulations. C, the number of human regulations identified from EVLncRNAs. D, the number of human regulatory divergent lncRNAs identified by IRDL, which located in human enhancer region and TF binding region. E, the number of of human regulations identified by IRDL that were validated by GTEx and LongHorn.

**Figure S5**

The number of predicted regulatory lncRNA-gene associations in a specific tissue/cell condition.

**Figure S6**

The comparison of the predictive performance on SVM and RF in terms of AUPR.

Table S1

|  | **gene** | **lncRNA** | **Specific tissue** | **enhancer** | **TF binding** |
| --- | --- | --- | --- | --- | --- |
| 1 | Foxd3 | Foxd3as | ESC |  |  |
| 2 | Evx1 | Evx1as | NA |  |  |
| 3 | Ccnyl1 | Ccnyl1as | brain | ✓ |  |
| 4 | Sox3 | Sox3as | brain |  |  |
| 5 | Nr2f1 | Nr2f1as | brain |  |  |
| 6 | Rab11b | Rab11bas | brain |  |  |
| 7 | Zfp687 | Zfp687as | brain | ✓ | ✓ |
| 8 | Gata3 | Gata3as | NA | ✓ | ✓ |

Table S2

|  | **gene** | **lncRNA** | **Specific tissue** | **loop validation** | **RISE** | | **enhancer** | | | **TF binding** |
| --- | --- | --- | --- | --- | --- | --- | --- | --- | --- | --- |
| 1 | Nkx2-3 | Gm20467 | intestine | ✓ | |  | | ✓ |  | |
| 2 | Bahcc1 | 2900052L18Rik | brain | ✓ | |  | |  | ✓ | |
| 3 | Maf | Gm15655 | brain | ✓ | |  | | ✓ |  | |
| 4 | 2300009A05Rik | Gm16759 | stomach |  | | ✓ | | ✓ | ✓ | |
| 5 | Hoxa3 | 2700086A05Rik | brain | ✓ | |  | | ✓ | ✓ | |
| 6 | Slc34a2 | Gm17182 | NA | ✓ | |  | | ✓ | ✓ | |
| 7 | Mdga1 | Gm16758 | NA | ✓ | |  | | ✓ | ✓ | |
| 8 | Baz2b | Gm13620 | brain | ✓ | |  | | ✓ |  | |
| 9 | Ugp2 | Gm12043 | muscle |  | | ✓ | |  |  | |
| 10 | B3gnt2 | 9130230N09Rik | brain | ✓ | |  | | ✓ | ✓ | |

Table S3

|  | **gene** | **lncRNA** | **specific tissue** | **RISE** | **loop validation** | **EVLncRNAs** | **LongHorn** | **GTEx** | **enhancer** | **TF binding** |
| --- | --- | --- | --- | --- | --- | --- | --- | --- | --- | --- |
| 1 | FOXF1 | FENDRR | lung |  | ✓ | ✓ |  |  |  | ✓ |
| 2 | ICA1 | AC007128.1 | blood |  | ✓ |  |  | ✓ |  | ✓ |
| 3 | BOK | BOK-AS1 | liver | ✓ |  |  | ✓ |  | ✓ | ✓ |
| 4 | MORC2 | TUG1 | blood |  |  | ✓ |  | ✓ |  | ✓ |
| 5 | ICMT | LINC00337 | ESC |  | ✓ |  |  | ✓ | ✓ | ✓ |
| 6 | RAD51 | RAD51-AS1 | blood |  | ✓ | ✓ |  |  |  | ✓ |
| 7 | SDR39U1 | LOC101927045 | liver |  | ✓ |  |  | ✓ |  | ✓ |
| 8 | TRAM2 | TRAM2-AS1 | liver | ✓ |  |  |  |  |  | ✓ |
| 9 | SLITRK5 | MIR4500HG | brain | ✓ | ✓ |  |  |  |  | ✓ |
| 10 | JAZF1 | JAZF1-AS1 | blood |  |  |  | ✓ | ✓ |  | ✓ |

Figure S1


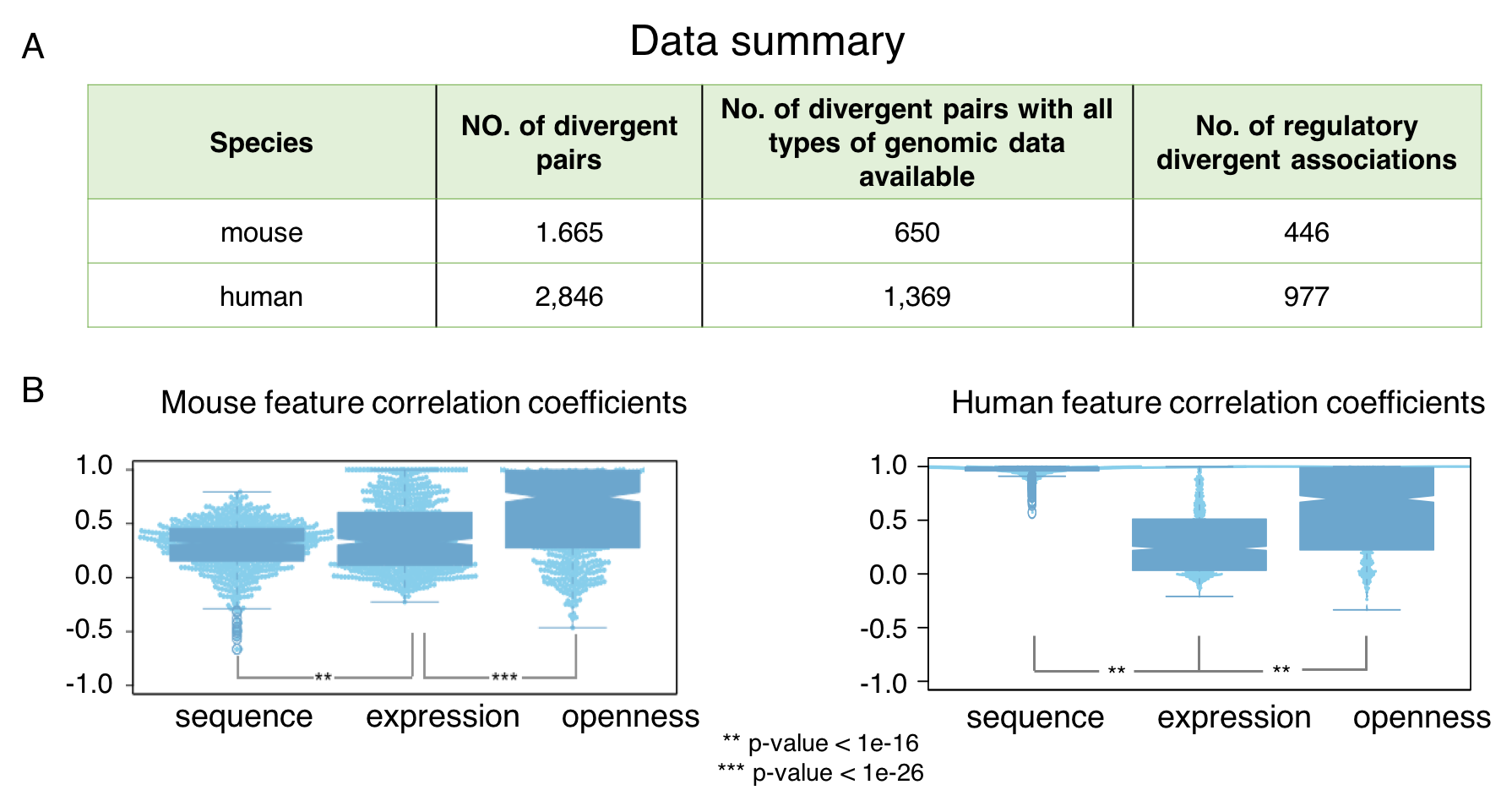


Figure S2


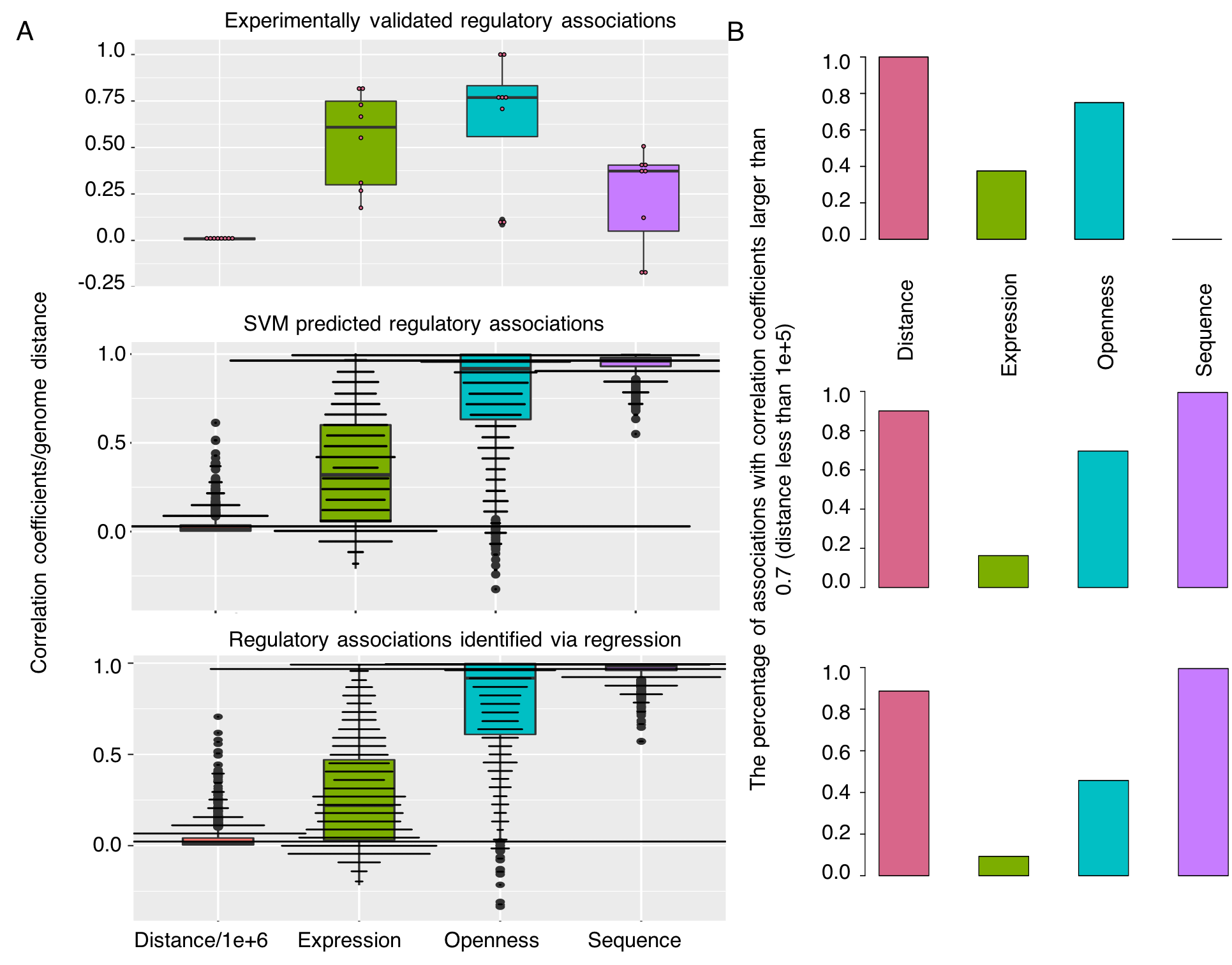


Figure S3


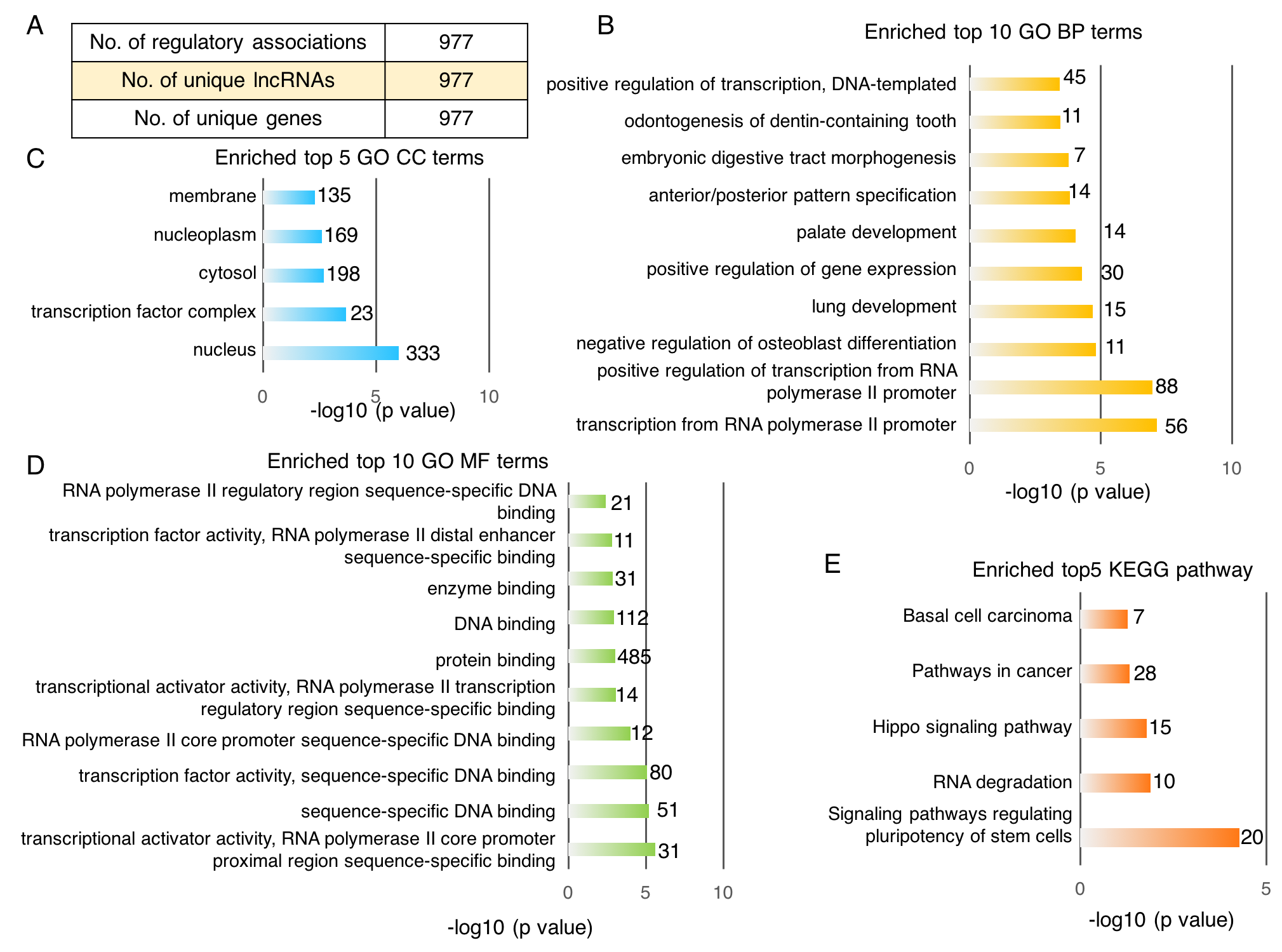


Figure S4


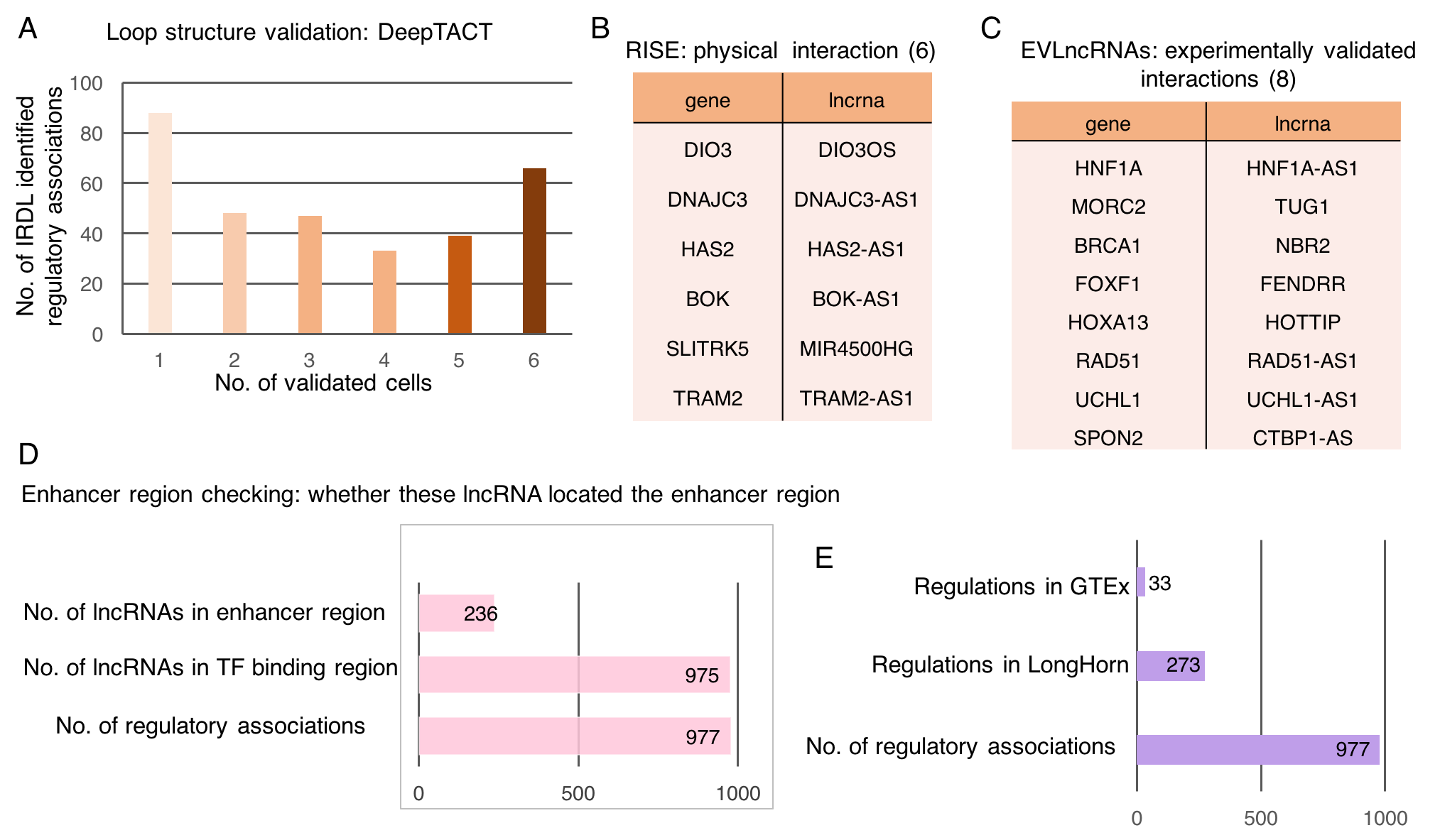


Figure S5


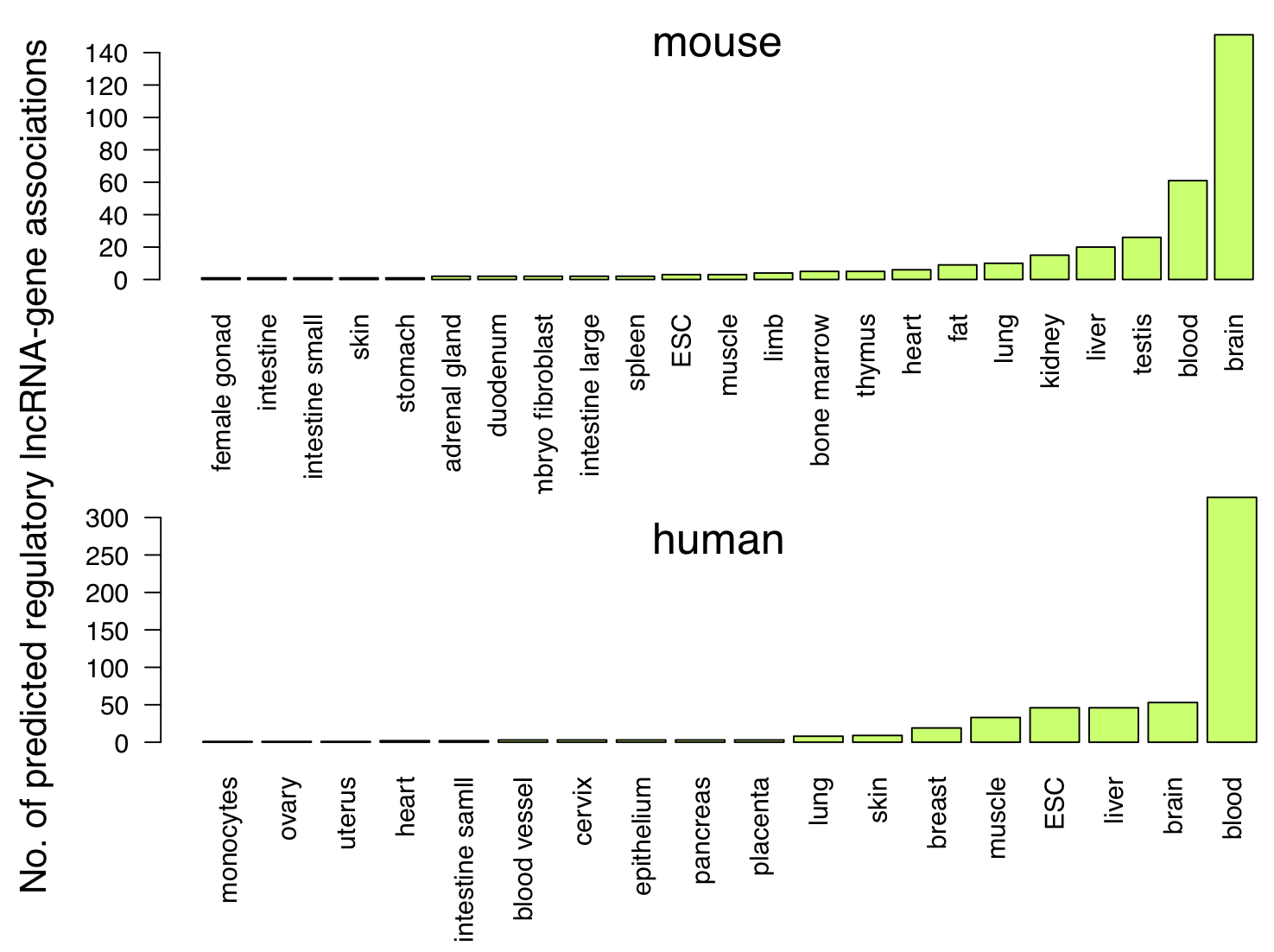


Figure S6
